# SynPAnal: Software for Rapid Quantification of the Density and Intensity of Protein Puncta from Fluorescence Microscopy Images of Neurons

**DOI:** 10.1371/journal.pone.0115298

**Published:** 2014-12-22

**Authors:** Eric Danielson, Sang H. Lee

**Affiliations:** 1 Department of Pharmacology and Toxicology, Medical College of Wisconsin, Milwaukee, Wisconsin, United States of America; 2 Neuroscience Research Center, Medical College of Wisconsin, Milwaukee, Wisconsin, United States of America; Virginia Tech Carilion Research Institute, United States of America

## Abstract

Continuous modification of the protein composition at synapses is a driving force for the plastic changes of synaptic strength, and provides the fundamental molecular mechanism of synaptic plasticity and information storage in the brain. Studying synaptic protein turnover is not only important for understanding learning and memory, but also has direct implication for understanding pathological conditions like aging, neurodegenerative diseases, and psychiatric disorders. Proteins involved in synaptic transmission and synaptic plasticity are typically concentrated at synapses of neurons and thus appear as puncta (clusters) in immunofluorescence microscopy images. Quantitative measurement of the changes in puncta density, intensity, and sizes of specific proteins provide valuable information on their function in synaptic transmission, circuit development, synaptic plasticity, and synaptopathy. Unfortunately, puncta quantification is very labor intensive and time consuming. In this article, we describe a software tool designed for the rapid semi-automatic detection and quantification of synaptic protein puncta from 2D immunofluorescence images generated by confocal laser scanning microscopy. The software, dubbed as SynPAnal (for Synaptic Puncta Analysis), streamlines data quantification for puncta density and average intensity, thereby increases data analysis throughput compared to a manual method. SynPAnal is stand-alone software written using the JAVA programming language, and thus is portable and platform-free.

## Introduction

The human brain is made up of billions of interconnected neurons that can each form thousands of connections (synapses) with other neurons. [1]–[3]. Abilities such as consciousness, emotions, intelligence, learning and memory are possible due to the complex interconnectivity between neurons [2], [4], [5]. Current models suggest that information storage occurs at synapses through structural and molecular rearrangements of synapses with multitudes of synaptic proteins being added to and/or released from the synapses [6]–[8]. Therefore, studying the dynamic reorganization and turnover of proteins at synapses is one of the hottest topics in the field of molecular and cellular neurobiology. Furthermore, in addition to learning and memory, these are directly relevant to understanding synapse/circuit development, mental retardation associated with aging and neurodegenerative diseases, and pathological bases of psychiatric diseases [9]–[12].

Immunofluorescence microscopy is one of the most widely used methods to investigate how synapses and the associated proteins contribute to various synaptic events. By measuring changes in puncta attributes such as density, size, and intensity of puncta, researchers are able to study how both the subcellular distribution and the localized levels of specific molecules change at synapses. For example, PSD-95 is a postsynaptic scaffolding protein frequently used as excitatory synapse markers. Changes in the puncta density and intensity of PSD-95 correlate directly with excitatory synapse numbers and synapse strength [13]. Therefore, puncta analysis is a crucial tool for understanding how molecular reorganization affects synaptic function. Unfortunately, quantification of synaptic protein levels in the fluorescent images is very labor intensive. Measurement of puncta attributes must be performed by manually counting and tracing each individual cluster along neuronal structures, such as dendrites and dendritic spines. Image analysis software, such as MetaMorph (Molecular Devices) and ImageJ [14], are typically used to perform these measurements. These programs are powerful tools for image analyses but are also designed to be versatile, so their use for performing specialized tasks, such as puncta analysis, can be cumbersome as data from different types of measurements must be reorganized and recombined for post-analysis using different software such as Excel. Another problem was that the software's automated particle detection algorithm would often detect multiple adjacent clusters as single objects, forcing user intervention or manual detection. Finally, these programs are not optimized for analysis of large image sets, and the detection settings (such as threshold) must be set and reset for each individual image, which increases the time spent analyzing images.

A number of software is available for the automated analyses of morphometric data of neurons, especially for dendritic spines [15]–[18]. However, to the best of our knowledge, only one attempt has been made to increase the efficiency of analyzing synaptic proteins by a software-based automated analysis: SynD [19]. SynD is a MatLab based program that automatically traces and measures dendrites of neurons labeled with a trace marker (typically space-filling GFP), and then quantifies the synaptic protein levels as a function of distance from the cell body. SynD's fully-automated strategy has been reported to increase the efficiency of analysis by approximately 90%. However, the reliance of SynD on cell-fill markers for the automatic selection of neuronal “regions” prevents the analyses of images lacking trace markers and/or soma (for example, high magnification images with only dendrites). Also, this prevents the comparison of synaptic protein levels in transfected neurons from those in neighboring non-transfected neurons, which serve as the best control. Furthermore, while SynD, excels at calculating the puncta density of proteins from the entire neuron it becomes less efficient at measuring and comparing fluorescent intensity when those measurements are restricted to specific sub-regions of the neuron.

In this report we present new software, named SynPAnal, specifically designed for the rapid analysis of 2D images of neurons acquired from confocal or fluorescent microscopy. The primary function of the software is the automatic and rapid quantification of puncta attributes, but in addition, the software also allows basic fluorescent intensity measurements, and allows for additional simple morphometric analysis of neurons, such as measurements of dendrite length, spine dimensions, spine categorization, and spine density.

## Materials and Methods

### Dissociated Neuron Culture and Immunocytochemistry

The use and procedure of vertebrate animals in this research has been approved by the IACUC of the Medical College of Wisconsin. For euthanasia of rats, CO_2_ gas administration was used, and death was assured by a secondary method of thoracotomy. Hippocampal culture neurons were prepared from rat embryos as described previously [20]. Neurons (div 14) were transfected with plasmids expressing β-galactosidase (β-Gal) or GFP using Lipofectamine 2000, fixed and processed for immunocytochemistry after 2 days post-transfection as described [21].

### Image acquisition

Images were acquired using a confocal laser scanning microscope (Nikon D-Eclipse C1). Typically 11 optical sections were scanned in with a width of 0.33 µm. Gain settings from each channel were adjusted to optimize signal to achieve less than 20% saturation from all puncta. Gain settings were kept constant for all images acquired from the same experimental trial.

### Image Conversion

Images acquired from Nikon D-Eclipse C1 produced an IDS file containing raw image data in 48-Bit RGB format and an ICS description file storing image dimensions and stack information. IDS 3D stack images were first converted into 24-Bit RGB format tiff images with maximal projection algorithm using a built-in Nikon EZ C1 software or custom software.

### Software Requirement

SynPAnal is a cross-flatform Java-based application, and thus operating system-independent. The software (SynPAnal installation.zip) can be downloaded from https://www.dropbox.com/s/ize5tvffdd22l9i/SynPAnal_Istallation_new%20version.zip?dl=0. To install the SynPAnal, Java runtime environment (version 8 or later) should be running in the system, which can be downloaded from http://java.com/en/download/index.jsp. The instructions for the installation and running of the software are described in detail in the SynPAnal User Manual (S1 Text). The software is designed to read 3 byte RBG uncompressed files of tiff, JPEG or GIF format images. Since Java does not natively support the manipulation of tiff image, SynPAnal uses the Java Advanced Imaging API for the analyses of tiff images. The sourced code is released under the General Public License, and is available upon request.

### Proof-of-principle Data Analysis

To compare the manual vs automatic quantification of puncta, eight images of neurons stained for PSD-95 and β-Gal were used. For dendritic spines analyses, images from twelve EGFP-transfected hippocampal neurons were used. Guides for dendrite regions were drawn for each image to ensure the same dendrite segments were analyzed by both methods. Automatic puncta quantification of puncta density and puncta intensity was performed using SynPAnal. Manual puncta quantification was performed using MetaMorph software (Molecular Devices, Inc). Statistical significance was determined using the Student's t-test (unpaired, two-tailed, assuming unequal variance). *p*<0.05 was considered significant.

## Results and Discussion

Manual analysis of synaptic proteins can typically take up to 10 minutes per image with a minimum of 8–15 images per condition. Therefore, for experiments involving multiple conditions, analyses of one set of images may take several hours. Because of this, a more efficient method of analysis was desirable. SynPAnal has been designed to automate the repetitive tasks and tedious work that accompanies manual quantification, while allowing the user to control precisely what regions get analyzed and what data is included.

### Semi-automated Puncta Detection

**Figure 1:**
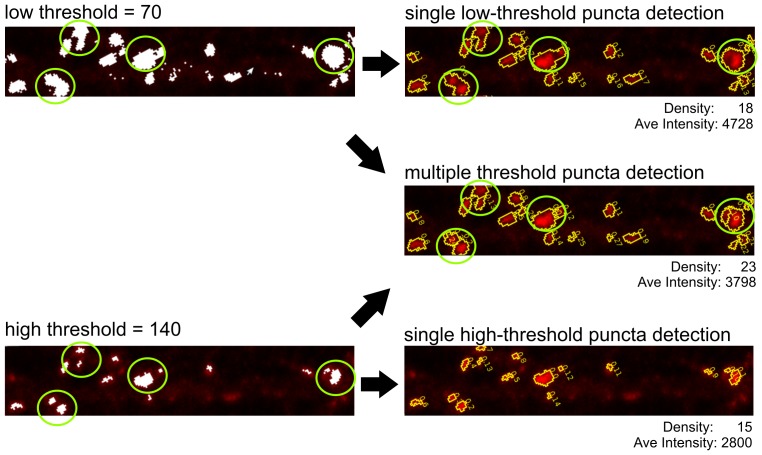
Improved puncta detection method of SynPAnal. An immunofluorescent image is shown to illustrate the differences between puncta detection at a single threshold versus puncta detection at multiple thresholds. A single round of puncta detection at a threshold of 70 returns 18 puncta with an average intensity of 4728 (top-right). At high threshold setting (140), 15 puncta were detected with an average intensity of 2800 (bottom-right). Green circles indicate multiple adjacent puncta that are detected as single clusters when using a low single threshold, but are actually two puncta at higher threshold setting (140), which resulted in an under-estimation of puncta numbers. SynPAnal uses multiple rounds of puncta detection starting at a threshold of 255 and ending at a threshold of 70 (center-right) and detected 23 puncta with an average intensity of 3798.

A major issue reducing analysis efficiency in ImageJ and MetaMorph was the need to manually count and trace puncta. Manual puncta detection is accomplished by thresholding images and manually locating and counting distinct clusters of 4 or more adjacent pixels above the threshold. The threshold setting is adjusted to a value that is high enough to exclude background noise while low enough to include as much immunofluorescent signal as possible. ImageJ and MetaMorph contain particle detection algorithms, but we found these algorithms inadequate as they could not be used to automatically calculate density and could only be applied to a single region of interest at a time. Additionally, the size and fluorescent intensity of the puncta can be highly variable depending on the protein being analyzed and the quality of the antibodies being used for identification. Often bright puncta that are in close proximity appear as a single large cluster when the threshold setting is low, artificially reducing the puncta density (Fig. 1). The particle detection algorithms in ImageJ and MetaMorph would often merge adjacent puncta into a single large cluster, artificially reducing the puncta density and increasing the average puncta intensity. To correct for this, large puncta need to be reexamined at higher threshold values so an accurate puncta density can be determined. This reexamination of large puncta increases the amount of time needed to analyze images.

Some of the tedium of puncta analysis can be circumvented by using automated software such as SynD, but there are certain situations where the use of SynD is not feasible. The first being when it was necessary to analyze neurons in the absence of a cell-fill marker. Additionally, it is sometimes necessary to analyze data exclusively from specific regions, such as spines, soma, proximal, or distal dendrites, which automated software may be unable to accomplish. We therefore decided to develop a semi-automated method that did not rely on the presence of a cell-fill marker and allows the user to designate specific area where the quantification occurs.

SynPAnal allows the user designate the areas of image from which puncta analysis should begin, by providing two dendritic area-drawing tools. The first tool automatically generates a fixed width rectangular segment around the dendrites. The second one allows the user to create a custom region surrounding the designated length of dendrite. The puncta in the designated area are then automatically detected based off the specified threshold settings. SynPAnal detects puncta by identifying clusters with four or more adjacent pixels above threshold. The algorithm circumvents the problem of merging adjacent puncta by performing multiple rounds of puncta detection, beginning at a high threshold value and finishing at a low threshold value (Fig. 1). During each round of detection, the threshold is lowered by one increment. Existing puncta are then expanded if adjacent pixels exist that exceed the current threshold value. Additionally, new puncta are formed if clusters of four or more adjacent pixels are above the threshold and are isolated from already existing puncta. Therefore, SynPAnal has improved puncta detection ability. The thresholding is used only to identify whether a pixel is a “part of a puncta” or not and is used to help distinguish adjacent puncta as distinct. The program calculates and reports both integrated intensity (the sum of pixel intensity values for each pixel in a puncta) and average intensity of each puncta (integrated intensity value divided by the number of pixels).

To ensure accurate puncta measurements, it is important to be able distinguish puncta from the neurons being measured and surrounding neurons. Often puncta adjacent but outside of the measured neuron are included. SynPanal contains features to exclude puncta from non-measured neighboring neurons. Firstly SynPAnal keeps the color channels for the analyzed image merged so the boundaries of the transfected neurons are clearly visible. Secondly, the custom-region dendrite allows the user precise control over which puncta are included in the analysis. Thirdly, the software can automatically remove puncta from the analysis if the puncta does not overlap with the transfection marker.

### Data Output

In MetaMorph and ImageJ, all of the data from the measurements from a single image is output in a spreadsheet style format. When analyzing neurons, there are multiple situations where this becomes inconvenient. The first is when data from different types of measurement must be combined in a specific manner. For example, to quantify the average puncta density from a neuron two types of measurement must be performed and combined; the length of multiple dendritic segments and the number of puncta in the corresponding segment. In ImageJ and MetaMorph, this is inefficient as it is then the user's responsibility to reorganize and recombine the raw data and then perform the necessary calculations. This reorganization process can be laborious and may introduce additional opportunities for mistakes. The second trouble we encountered that reduced efficiency was when it was necessary to analyze two different attributes from the same image simultaneously. When the data is lumped together the user must either perform each analysis separately or keep track of what row, in the spreadsheet, corresponds to each attribute. This is especially evident when attempting to simultaneously measure spine density, puncta density and puncta intensity.

**Figure 2:**
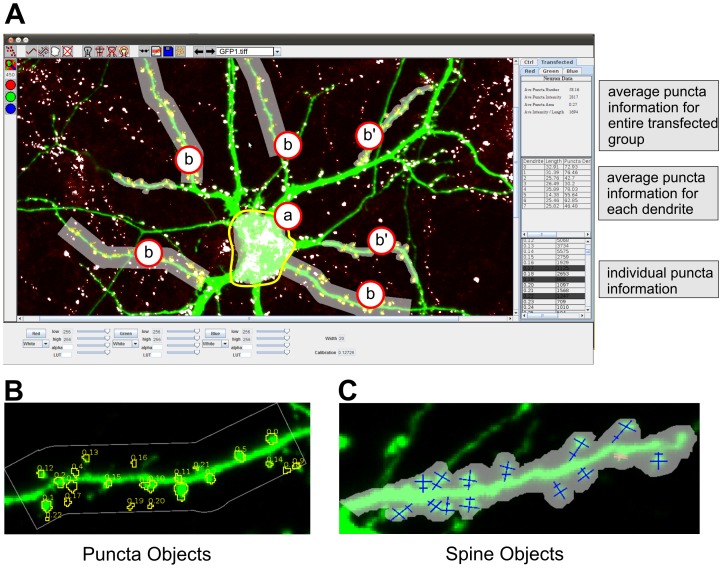
Screenshot of SynPAnal with neuron-themed objects. **A**) Screen shot of image analysis being performed with SynPAnal. Cell body (a) object used to measure the fluorescent intensity from the soma of neurons. Dendrite objects (b and b') are used to quantify puncta density and intensity from transfected neurons. Puncta data is displayed in the data panel on the right of the program. The bottom panel shows the puncta information for each individual puncta. The middle panel shows the average puncta information for each dendrite. The top panel shows the average puncta information for the entire transfected group. **B**) An isolated view of a dendrite object with the contained puncta measuring objects. **C**) An isolated view of a dendrite object with contained spine measuring objects. For complete description of all features of the software, see SynPAnal User Manual (S1 Text).

To circumvent these problems, SynPAnal contains a variety of neuron-themed digital objects that perform specific types of neuron analysis to better organize data and make the analysis process easier (Fig. 2A–C). SynPAnal uses four digital objects to perform three specific types of analysis: puncta analysis, spine analysis, and general fluorescent intensity analysis. The first object is the “generic” object. This object allows the user to select a region of the image from which general fluorescent intensity analysis will be collected (Fig. 2 A, a). These objects automatically calculate the integrated intensity of a region or the average fluorescent intensity of a region. The second object is the “dendrite” object (Fig. 2A, b and b'). The dendrite allows the user to specify a short region of dendrite to be analyzed. There are two types of dendrite object: the first automatically generated a fixed width rectangle surrounding the dendrite (Fig. 2A, b), the second allows the user to specify the region surrounding the dendrite in a “freehand” manner from which the fluorescent intensity measurements will occur (Fig. 2A, b'). These objects are used to calculate the length, total fluorescent intensity and average fluorescent intensity within the region containing the dendrite. In addition, dendrite objects serve as containers for the third and fourth objects the “puncta” and “spine” objects (Fig. 2B,C). The puncta objects are automatically generated and will calculate the integrated intensity and area of each puncta. The spine objects are manually placed and can act as markers for spine location or can be used to perform manual measurement of spine head width, spine length and spine neck width. In addition, the spine objects will use the spine morphology measurements to automatically classify the type of spines (mushroom, thin, stubby, or filopodia) [20], [22]. Alternatively, the user can also specify the spine type manually. The dendrite objects use the information from their contained puncta objects to calculate the puncta and spine density, average puncta intensity, puncta number and total puncta intensity per length. In addition to the aforementioned calculations, the dendrite objects use the information from their contained spine objects to calculate spine density, average spine-head width, spine length, and average spine density separated by spine-type. The dendrite objects can also use the information from both the spine and puncta objects to calculate the number of puncta per spine. We recognized that depending on the experiment, the user may need the data from each individual object, may need the data averaged by dendrite or may need the values averaged for the entire neuron. SynPAnal performs all these calculation automatically and the user specifies how the data will be written to a text file.

Data management can be problematic when multiple attributes from a single image are measured. To improve data management, the software allows the user to create categories to which each of the digital objects can be added so that the same type of object can be used to measure different types of data. For example, it is common to analyze the fluorescence from transfected and non-transfected cells within the same image. In SynPAnal, the user can create two groups: one for the transfected data and one for the non-transfected data then can add dendrite objects to each group to measure the puncta density from transfected and non-transfected neurons from the same image simultaneously while keeping the data from each category separate.

SynPAnal's digital objects reduce the amount of time needed post-analysis to perform additional calculations because the majority of the calculations have been performed already. The use of digital objects also allows two or more attributes to be easily analyzed simultaneously as the data is automatically organized by the desired attribute. Finally the ability to create different categories to organize the analysis allows the user to simultaneously analyze the same attribute from different areas or from separate populations of cells on the same image.

### Analysis for Large Image Sets

To increase efficiency in SynPAnal, the threshold settings from the previous image are automatically applied to the newly opened images, all digital objects are automatically saved and loaded and the data is automatically exported so that the user is only required to focus on analyzing the images.

### Proof-of-principle Analyses

**Figure 3:**
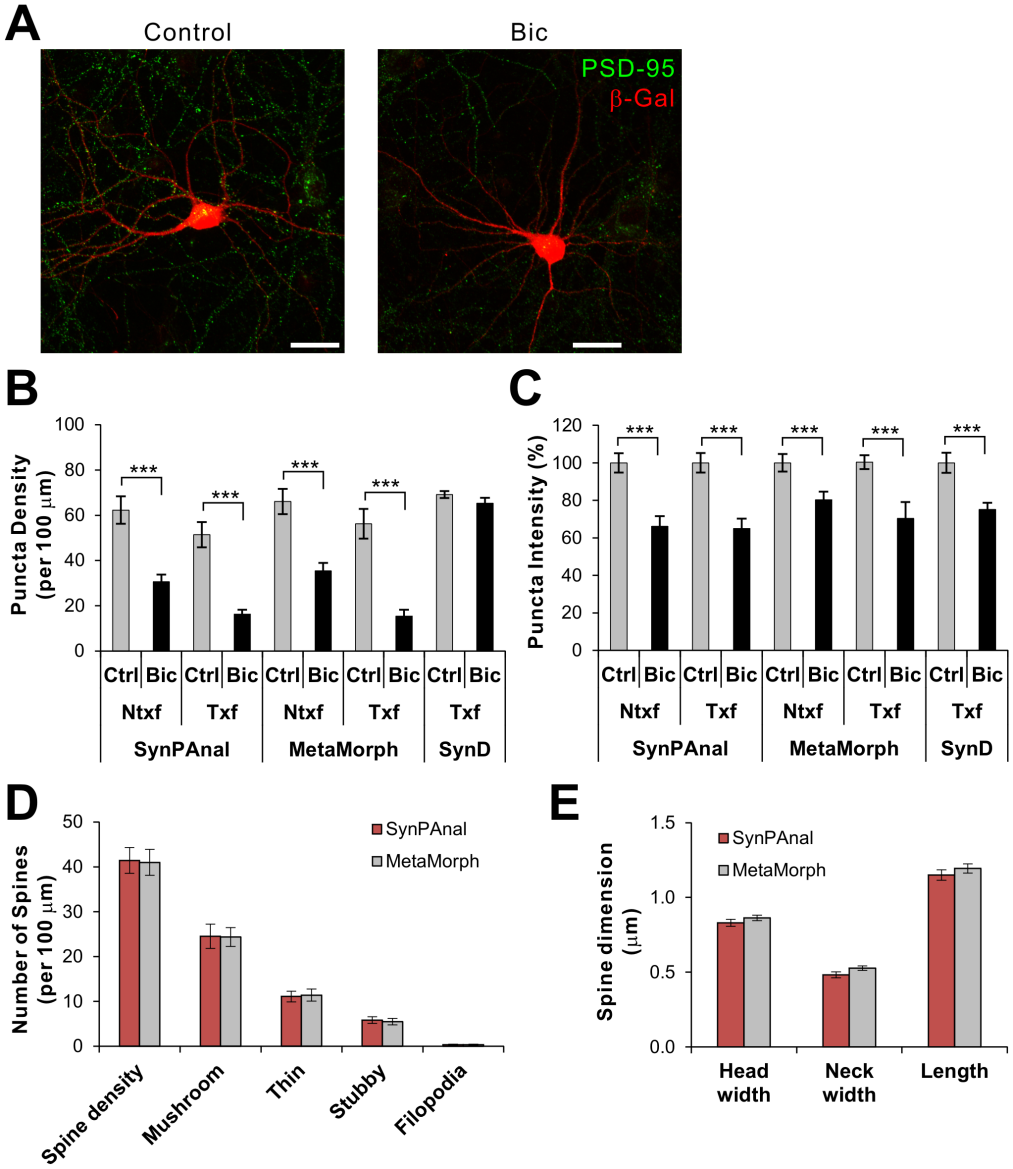
Comparison of the results of puncta and dendritic spine quantification from SynPAnal and MetaMorph. **A**) Representative images of hippocampal neurons stained for PSD-95 (green) and β-Gal, which were either mock treated (Control) or 40 µM Bic. **B, C**) Quantifying puncta density (**B**) and intensity (**C**) with SynPAnal produces similar results to MetaMorph. *n*>12 per condition, *** *p*<0.001. **D**, **E**) Dendritic spine data analyses for the density and types of spines (**D**) and dimension of spines (**E**) using SynPAnal and MetaMorph. Spine density was measured from 5 dendritic segments for each neuron (*n* = 14). The same dendrites were analyzed for both software. Approximately 40-70 spines were analyzed for each neuron.

To test the accuracy and efficiency of puncta analyses by SynPAnal, we compared the results from the program with those obtained from manual analysis using MetaMorph and fully-automatic analysis done by SynD program. For this comparison, we used a set of immunofluorescent confocal images taken from hippocampal neurons, which were transfected with β-galactosidase (β-Gal) and immunostained for endogenous PSD-95 (Fig. 3A). To compare the effectiveness of quantifying puncta changes under different conditions, we also analyzed images from the neurons treated with bicuculline (Bic, 40 µM) for 48 h before immunostaining, which should reduce both the intensity and density of PSD-95 puncta [21]. The results from SynPAnal's analyses of the PSD-95 puncta were comparable to those from the manual analysis method (MetaMorph) in both density and intensity (Fig. 3B,C), demonstrating the reliability and accuracy of our software. Furthermore, both analyses showed similar reduction of PSD-95 puncta density and intensity by Bic treatment. In contrast, SynD analyzed data showed the similar numbers in puncta intensity measurements but failed to detect the Bic-induced reduction of puncta density. The reason for the failure is unclear but presumably due to the difference in the threshold settings (SynD does not return the automatically determined threshold value). The most beneficial aspect of SynPAnal was its efficiency. In our hands, the software greatly reduced the amount of time spent analyzing each images, comparable to the throughput of fully-automatic SynD. We also analyzed a set of images for dendritic spine quantification using SynPAnal and MetaMorph. Both software returned almost identical results for the dendritic spine density, type of dendritic spines (mushroom, thin, stubby, and filopodia) (Fig. 3D). Furthermore, analyzed spine dimension data were undistinguishable between the two programs (Fig. 3E).

### Future Directions

Currently the functions of SynPAnal are limited to synaptic puncta analysis, fluorescent intensity analysis, and spine dimensional analysis of 24 bit RBG images. In the future, we plan to continue improving the software by expanding SynPAnal's capabilities and image compatibility. Incorporations of new features such as puncta co-localization measurement, incorporation of a puncta quantification feature into the generic regions, particle tracking, and microtubule categorization are under development. Furthermore, as the software is open-sourced, we welcome the inputs from the science community for any suggestions and modifications.

## Conclusions

Accurate measurement of protein levels at synapses is a crucial tool for understanding how synaptic proteins contribute to synaptic signaling. Our software aims to augment this tool by increasing the efficiency while retaining the accuracy of analysis. Besides an increase in efficiency, our software provides additional advantages. Firstly, the software is JAVA based and, therefore, can be utilized on Windows, Mac, or Linux systems. Secondly, the software is small (∼5 MB) in size and easily portable. Lastly, the software is freely available for use in any academic lab.

## Supporting Information

S1 Text SynPAnal User Manual.
(PDF)
