# Correction: SynPAnal: Software for Rapid Quantification of the Density and Intensity of Protein Puncta from Fluorescence Microscopy Images of Neurons

**DOI:** 10.1371/journal.pone.0118657

**Published:** 2015-03-05

**Authors:** The PLOS ONE Staff

In the Materials and Methods section of the article, in the second sentence of the Software Requirement subsection, the link from which SynPAnal can be downloaded is incorrect. The correct link is: http://figshare.com/articles/SynPAnal_Program/1285116. The correct second sentence is as follows: The software (SynPAnal installation.zip) can be downloaded from http://figshare.com/articles/SynPAnal_Program/1285116.
