# Supplementary material for: SynPAnal: Software for Rapid Quantification of the Density and Intensity of Protein Puncta from Fluorescence Microscopy Images of Neurons
Source: PLoS One. 2014 Dec 22;9(12):e115298. doi: 10.1371/journal.pone.0115298 (PMC4274056; doi:10.1371/journal.pone.0115298)

SynPAnal User Manual

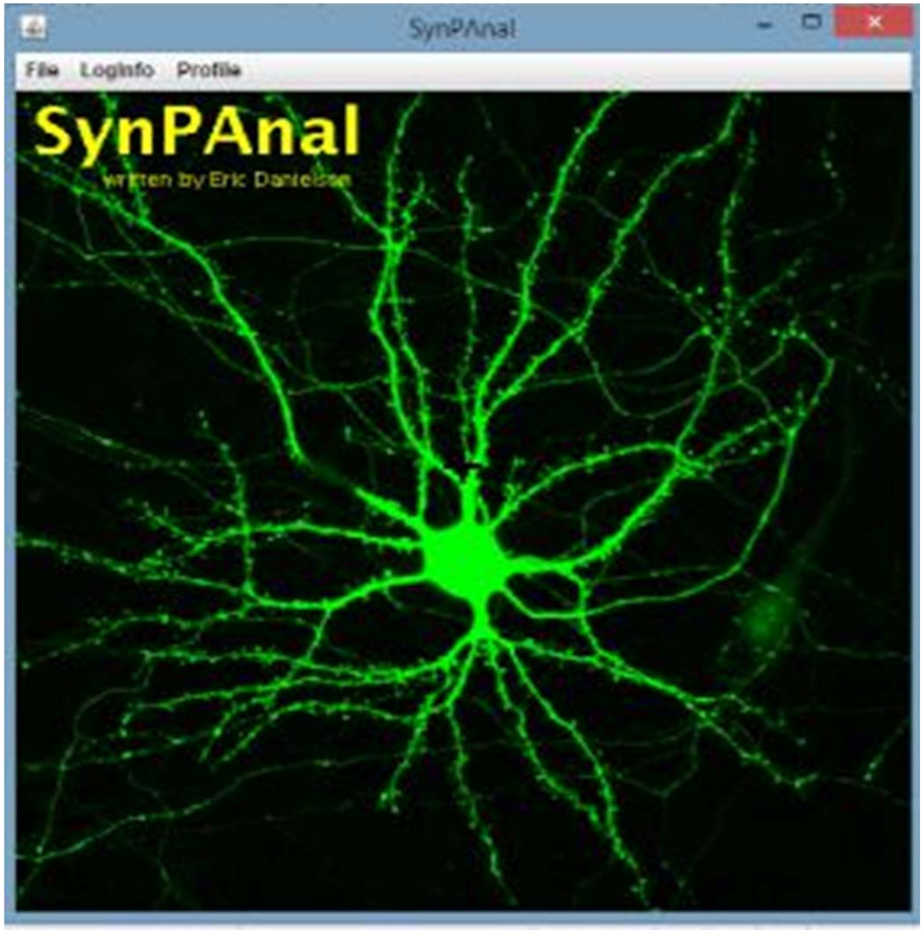

Eric Danielson and **Sang H. Lee**
Medical College of Wisconsin

Installation Instructions

Make sure the most recent version of JAVA is installed and is functioning. Unzip the SynPAnal_Installation.zip file into a new directory. A SynPAnal.jar file and directories “ext” and “profiles” should be created. Double click the SynPAnal.jar file to run the software.

Software Overview

SynPAnal is designed to aid the quantification of puncta from within the dendrites of neurons.

The steps of image analysis are as follows:

1) The user starts the software and opens all images to be analyzed. All images should be stored in the same directory and should be 8-bit RBG color TIFF files. SynPAnal can open 16-bit images but will convert them to 8-bit images.

2) The user should choose what data should be written, where the data should be written and from what color the data should be collected.

3) The user should then set the desired threshold. Once the threshold is set it will be saved and reloaded. The current threshold setting will be applied to all subsequent opened images unless the images have been thresholded previously, in which case the saved threshold will be loaded.

4) The user will then choose what regions will be analyzed by drawing either dendrite objects or general region objects onto the image. Puncta will automatically be quantified within the dendrite objects. Undesired puncta can be “removed” by right-clicking the puncta to clean up any errors.

5) The user then proceeds to the next image, All region information is saved automatically, all data is written automatically and the current setting are applied to the next image (unless already analyzed).

6)The user continues in this fashion until all images are analyzed. Following analysis, data is copied and pasted from the data text files and into excel for further analysis.

Introductory Screen

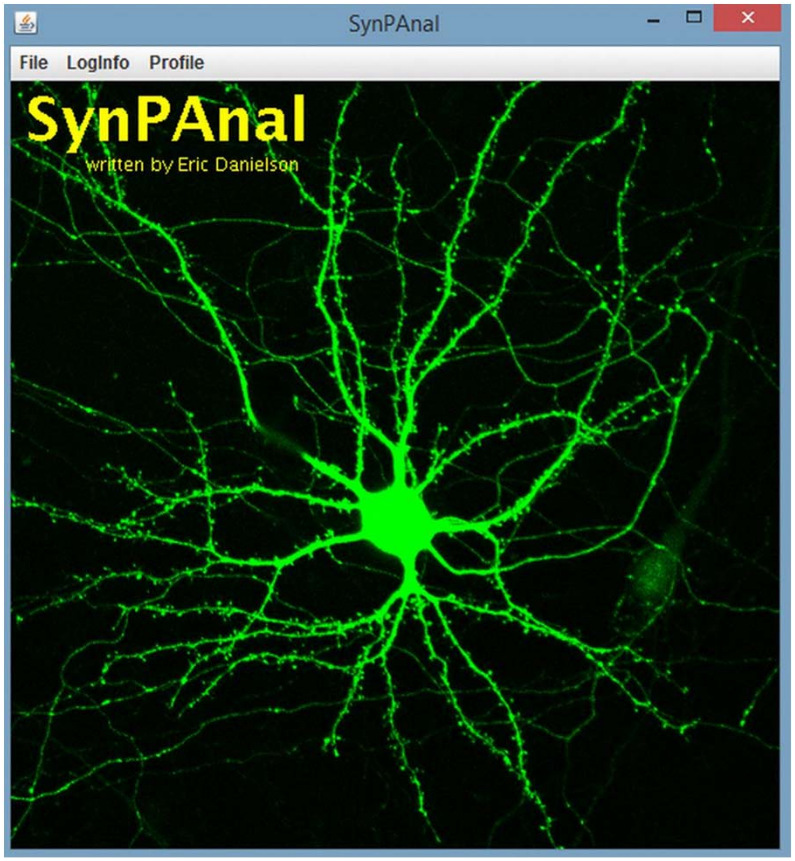

Introductory Screen
Starting the software will bring up the introductory screen containing a menubar with 3 menubar items: File, LogInfo and Profile.

File Menu
-Open: Allows the user to select what images will be analyzed.
-Change Log Directory: Allows the user to select the directory where the data will be written. The default directory is the directory that contains SynPAnal.
-Close: Exits the software.

LogInfo
-Data Output Colors: Allows the user to select data from what color channel will be output to a file.
-Data Output Type: Allows the user to select what type of data will be written to a file. The LogInfo section contains more information about these options.

Profile
-Calibration information can be stored into profiles and loaded by selecting the desired profile. Future versions of the software may contain more complex information in profile files.

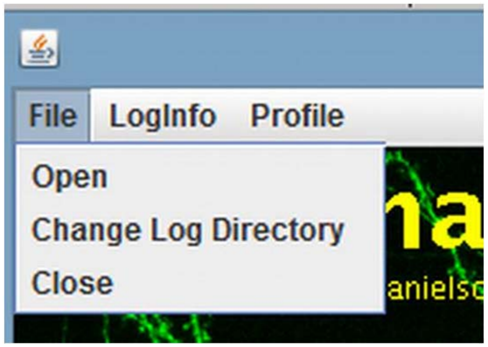

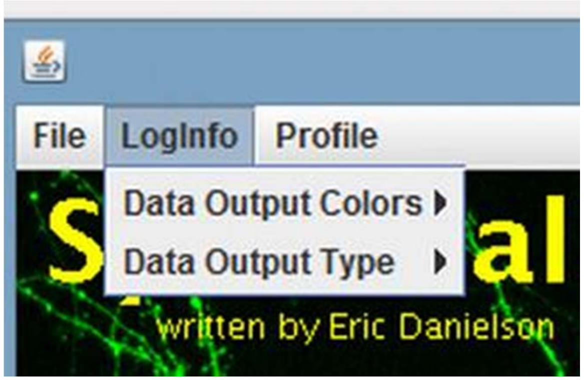

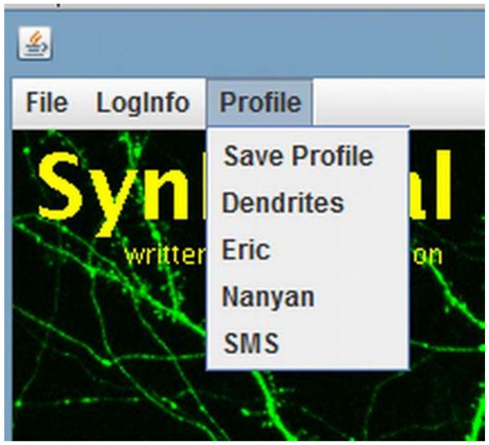

Data Logging

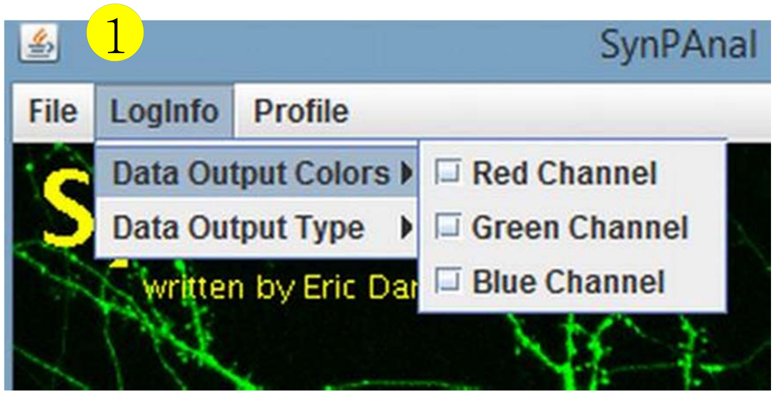

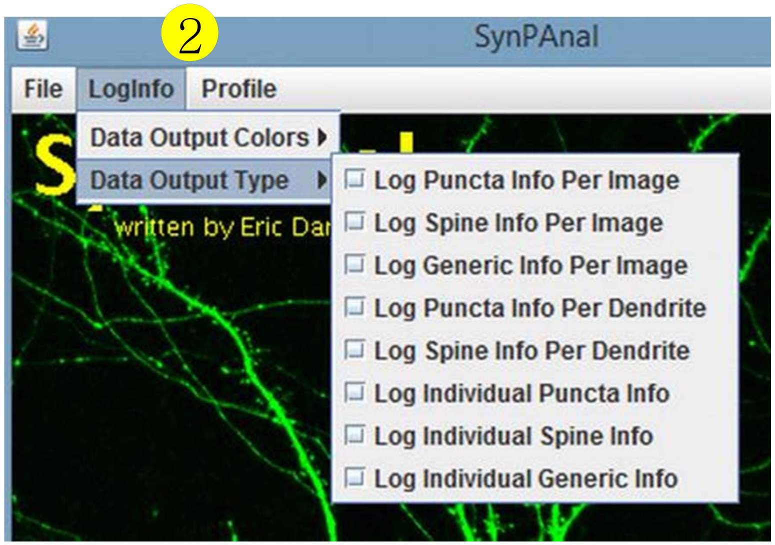

Data Output Colors (1)
Before data can be written to a file the user must select the channel from which the data measurements will be collected. 0-3 channels can be selected. *Known bug for spine dimension analysis a color must also be selected.

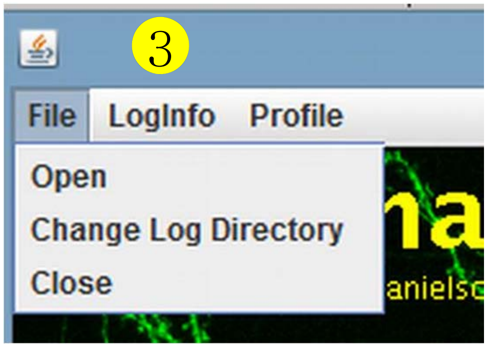

Data Output Type (2)
Before Data will be written to a file the user must select what data will be written. Any number can be selected at a time.

Change Log Directory (3)
The default directory where the data will be written is the directory containing SynPAnal.jar . The user can choose a different directory if desired.

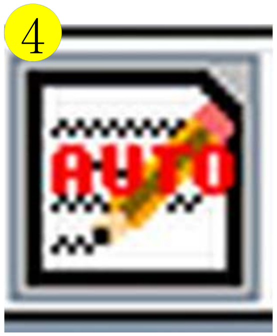

Auto Data Logging (4)
Left clicking the Button (4) will manually write data to a file. Generally this is not needed as data is written to a file as the user progresses to the next image. (This feature was included if only one image was being analyzed). Additionally the user can deactivate Auto Data Logging by right clicking button (4). The Auto text will disappear from the button. (This feature was included so users could go back to previous images without duplicating the data written to a file.

Analysis Screen

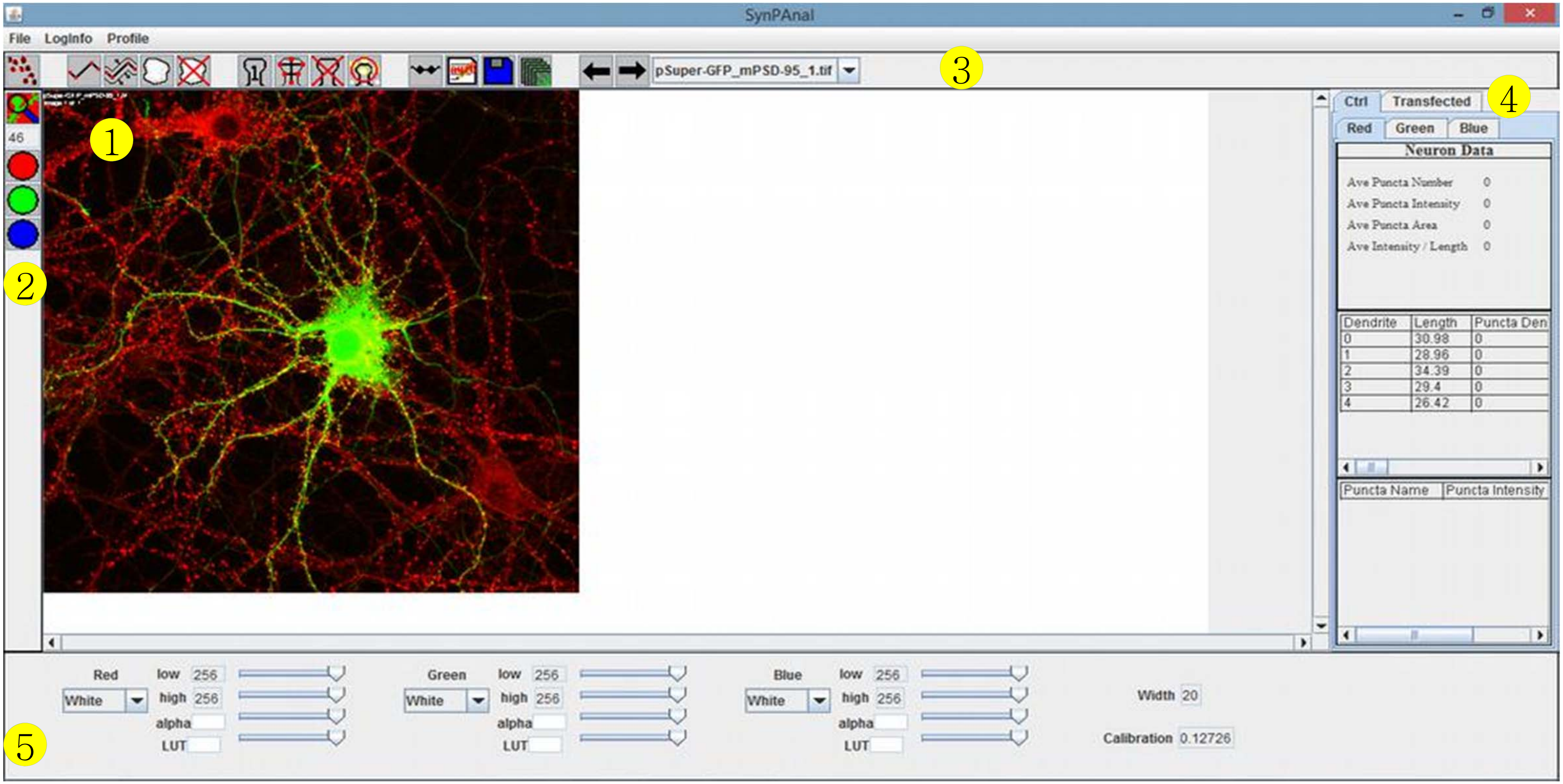

1 Image Panel: Draws image, threshold overlay and displays region objects where data measurements will occur

2 Zoom Panel: Controls image magnification and display of red green and blue color channels

3 Button Panel: Contains buttons for creation of measurement tools, writing data to a file and for controling navigation to and from images

4 Data Panel: Displays current data

5 Thresholding Panel: Displays tools for adjusting image thresholding, threshold overlay display and image channel look up tables

Zoom Panel

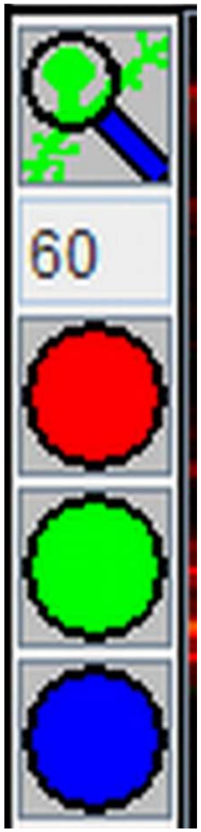

Toggles “Zoom” mode. Zoom in and out controlled by mouse wheel or “+” and “-” keys

Text box displaying zoom factor (adjustable)

Toggles whether red channel is displayed or hidden

Toggles whether green channel is displayed or hidden

Toggles whether blue channel is displayed or hidden

F-Key Button Panel

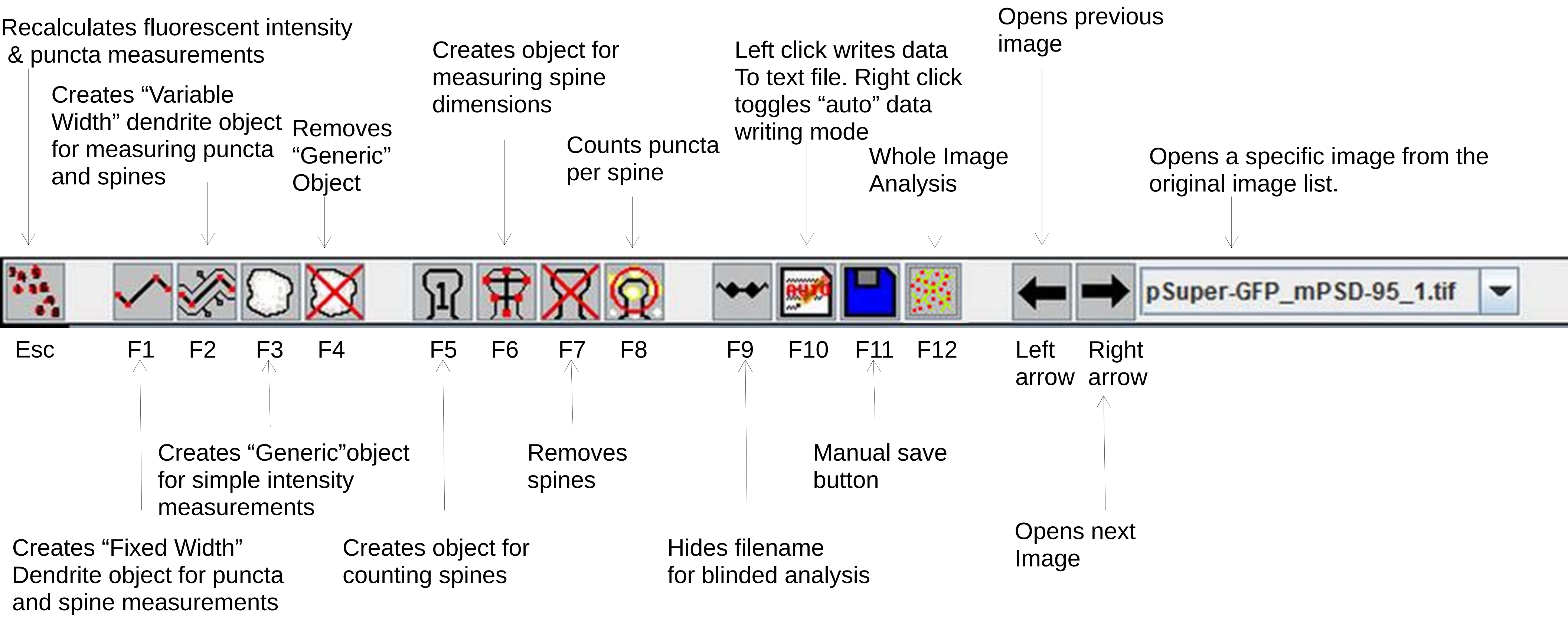

These buttons control:

1) The addition/removal of regions used for performing measurements.
2) Saving and writing data to a file
3) Navigation to and from images.

The layout of the buttons is supposed to mimic the F-Key layout on a standard keyboard. Esc, F1-12 and left and right arrows also trigger the button function

Thresholding Panel

Red threshold controls   Green threshold controls   Blue threshold controls

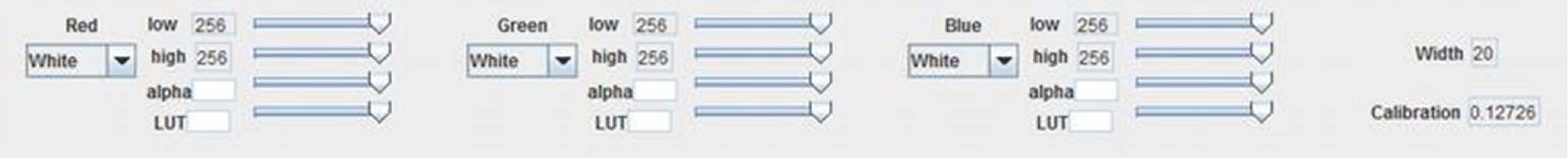

Channel to be thresholded   Threshold value (adjustable)

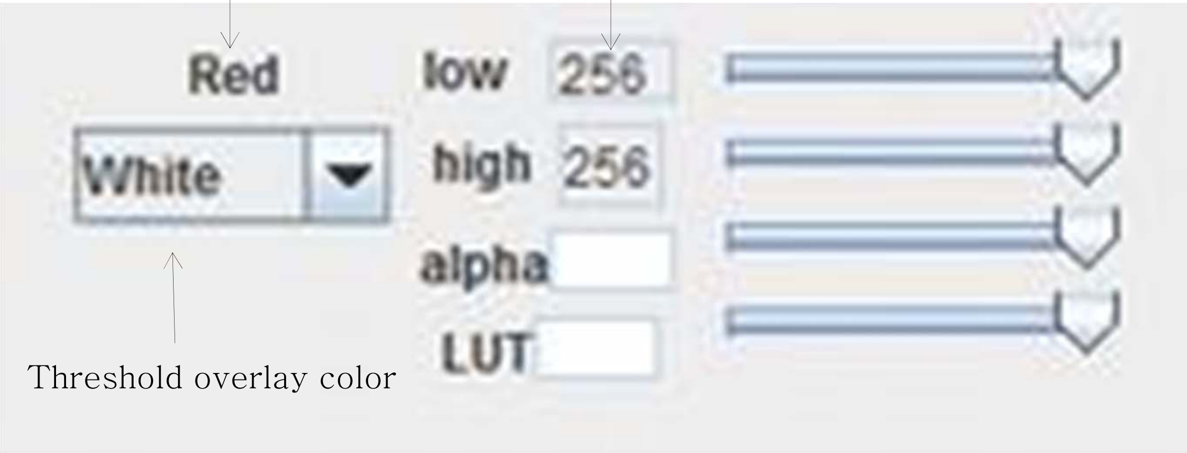

Low threshold slider

High threshold slider

Threshold overlay transparency slider

Image look up table slider

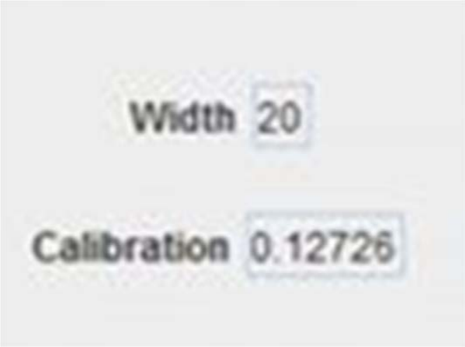

“Fixed Width” dendrite width. (adjustable)

Calibration factor: microns per pixel (adjustable and can be loaded from profile information)

Data Panel

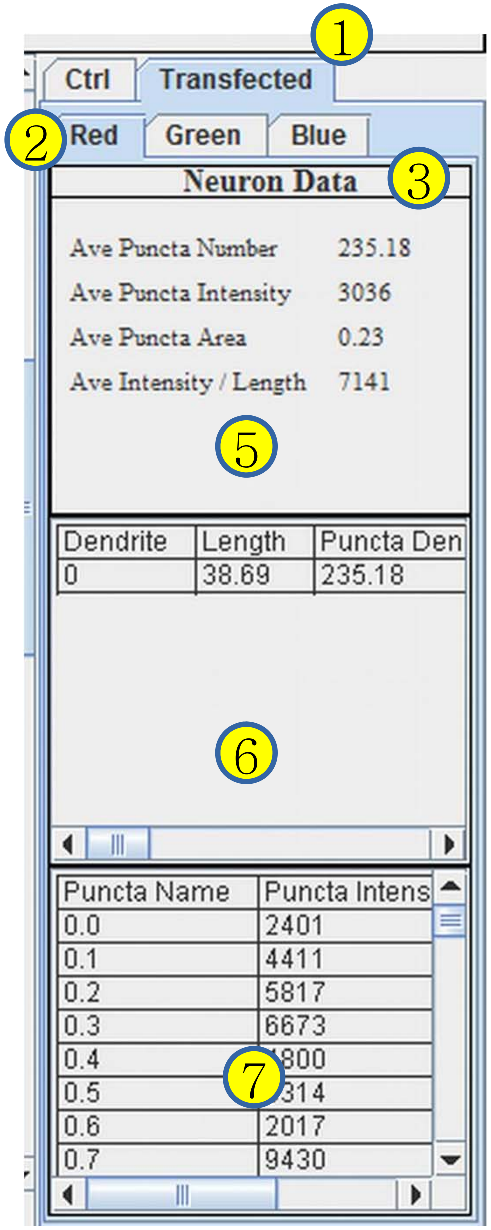

Group Tab Panels
The tab panels will allow you to switch between groups of objects that belong to each tab. It will also display information about measurements taken by those objects. In the left example two tabs have been set up. “Ctrl” and “Transfected” (1). The user can click on the one of the tabs to highlight it. Currently the “Transfected” tab (1) is highlighted. When this tab is highlighted the user can create and modify objects in this tab group. e.g. Dendrite objects added to the image when the “Transfected” tab is highlighted will be hidden when the “Ctrl” tab is highlighted. Additionally data from each tab group will be calculated, displayed and output separately. This will allow for separate populations i.e. “Transfected” and “Non-transfected” neurons to be analyzed in the same image.

Color Tabs
The user can click on “Red”,”Green” or “Blue” sub-tabs(2) to display color specific information.

Data Types
The user can click on “Neuron Data” (3) to cycle between “Puncta Info”, “Spine Info” and “Cell region Info”

Data Panels
The Neuron Panel (5) contains information averaged per image. The Dendrite Panel (6) contains information averaged per dendrite. The Individual Panel (7) contains the individual puncta, spine or generic region object information.

Adding and Removing Tab Panels
New groups can be created by right clicking the group tab and choosing add tab(8). Deleting a tab removes the tab and all regions associated with it. (This cannot be undone)

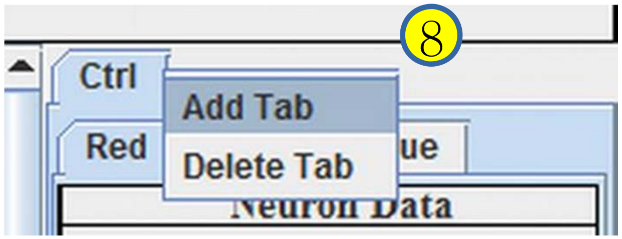

Dendrite Objects

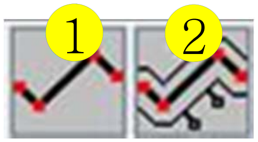

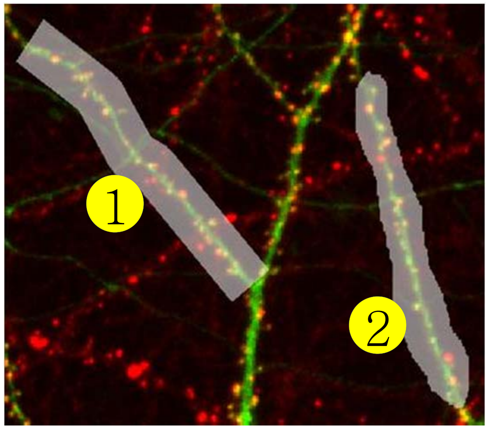

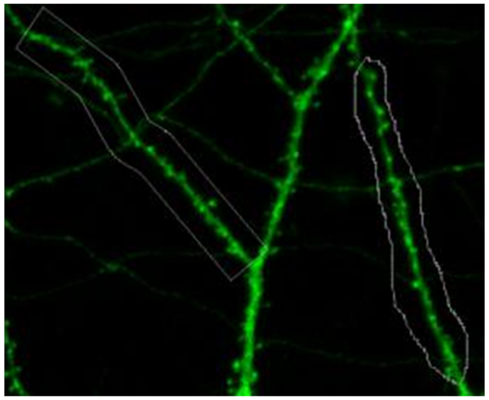

Fixed Width Dendrites

Clicking on button (1) or pressing "F1" will activate "Add Fixed Width Dendrite" mode. Once this mode is activated left clicking anywhere on the opened image will add a vertex to the dendrite object. Right clicking will remove vertices. The user should use this mode to add vertices to the center of the dendrite to be traced. Double left clicking will add the final vertex and will create a fixed width dendrite. Width is altered by changing width value in the Threshold Panel. All fixed width dendrites will have the same width. Addition of a dendrite will also trigger the automatic counting of puncta.

Variable Width Dendrite

Clicking on button (2) or pressing F2 will activate "Add Variable Width Dendrite" mode. As with the fixed width mode left clicking and right clicking will add and remove vertices and double clicking will add the final segment. After doubling clicking the user will hold down the left mouse button to trace the desired region around the dendrite. Double clicking will finish tracing the region.

Dendrite View

Pressing the V key cycles through a solid and open dendrite view mode.

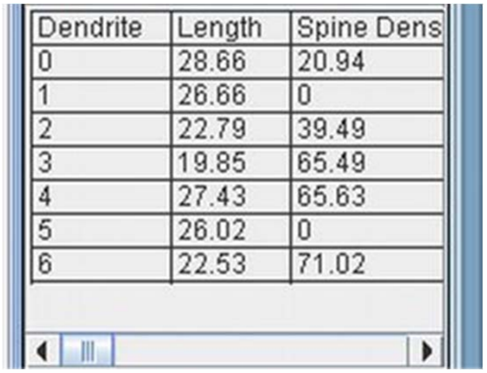

Dendrite	Length	Spine Dens
0	28.66	20.94
1	26.66	0
2	22.79	39.49
3	19.85	65.49
4	27.43	65.63
5	26.02	0
6	22.53	71.02

Dendrite Data Panel

Measurements from dendrites can be excluded from the analysis by "ignoring" specific dendrites. This is done by right clicking the desired dendrite row in the Dendrite Data Panel. Ignored dendrites will be colored a dark grey in the data panel. Left clicking the dendrite row in the Dendrite Data Panel will bring that dendrite into view in the main image.

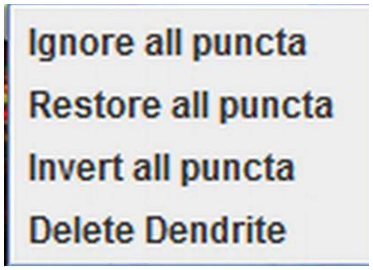

Right Click Options

Holding down the right mouse button over a dendrite brings up various options. Ignore all/Restore all puncta will exclude/include all puncta within the dendrite from analysis. Invert all puncta will swap which puncta are included/excluded. The delete dendrite object will permanently delete the dendrite and all puncta.

Puncta Objects

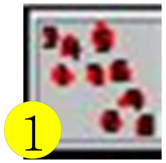

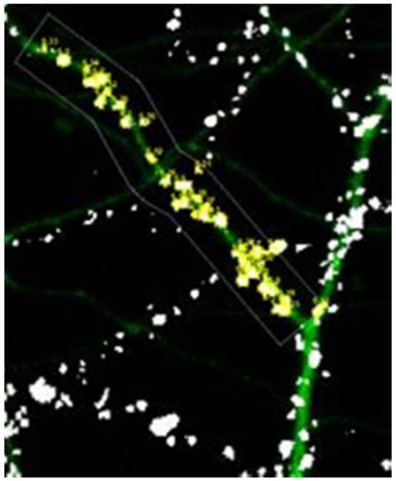

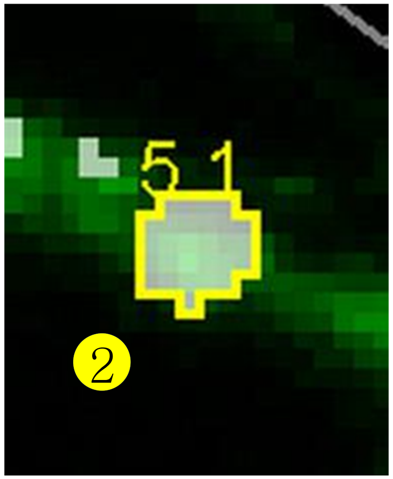

Counting Puncta

Clicking on Button (1) or pressing Esc key will automatically add puncta objects within drawn dendrite objects. A yellow border will be drawn around all puncta objects and the puncta lable drawn adjacent to the puncta. (2) Puncta are labeled as “X.Y”: X indicates which dendrite the puncta belongs to and Y indiciates the order in which the puncta was found. Puncta cannot be deleted but can be excluded from the measurement by right clicking the puncta object or the puncta row in the puncta data panel. To count puncta dendrite objects must be present and the image must be thresholded for the desired color channel.

Puncta Data Panel

Puncta data is displayed averaged per image (3), per dendrite (4) or for each individual puncta (5). Ignored puncta rows are colored dark gray. Values from ignored puncta do not contribute to the final totals and are not output to a file.

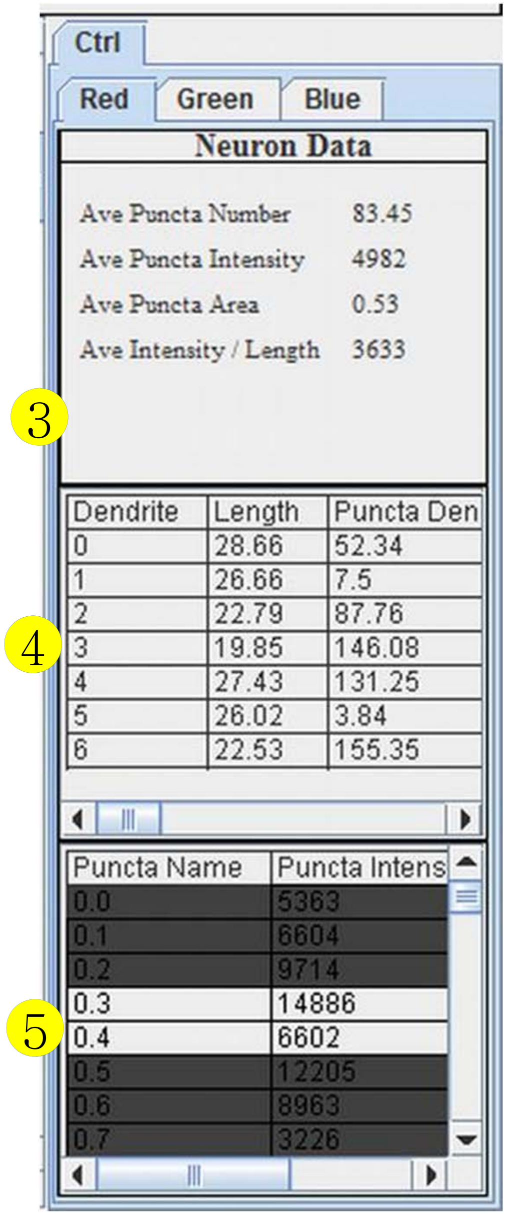

Spine Objects

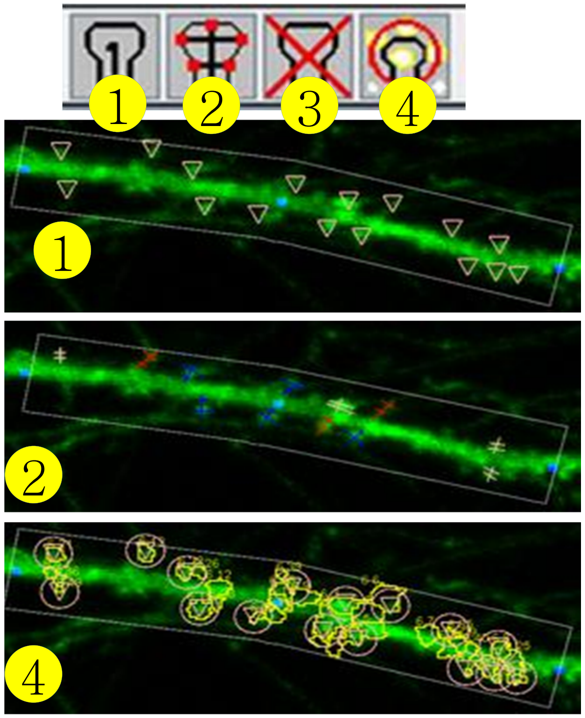

Right Click Options
Holding down the right mouse button over a spine will allow you to manually specify the spine type.

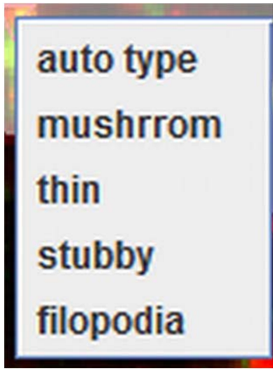

Counting Spine Objects
Clicking on Button (1) or pressing F5 will allow the user to add counting spine objects. The user can continue adding spines until finished. To end “Add Spine” mode the user should left click anywhere outside of a dendrite object. This mode adds spines quickly and is exclusively for calculating spine density

Measuring Spine Objects
Clicking on Button (2) or Pressing F6 will activate “Measure Spine” mode. In this mode 3 segments will be added in this order “Spine length”, “Spine Head Width” and “Spine Neck Width”.These measurements will be used to categorize the spines into Mushroom, Thin, Stubby and Filopodia. Mushroom spine will be drawn blue, thin red, stubby pink and filopodia yellow. Spine density will also be calculated

Punca per spine mode
Clicking on Button (4) or pressing F8 will allow the user to automatically remove puncta that do not fall within a specific radius of the spine. Pgup and pgdn increase or decrease the radius. The number of spines with 0, 1, 2 or $\geq$ 3 puncta will be calculated.

Deleting Spines
Clicking Button (3) or F7 activates “Remove Spine”. Clicking on spines during this more removes spines. Removing a spine deactivates this mode.

Spine Data Pane
Spine Data is averaged per Image, per dendrite or individually.

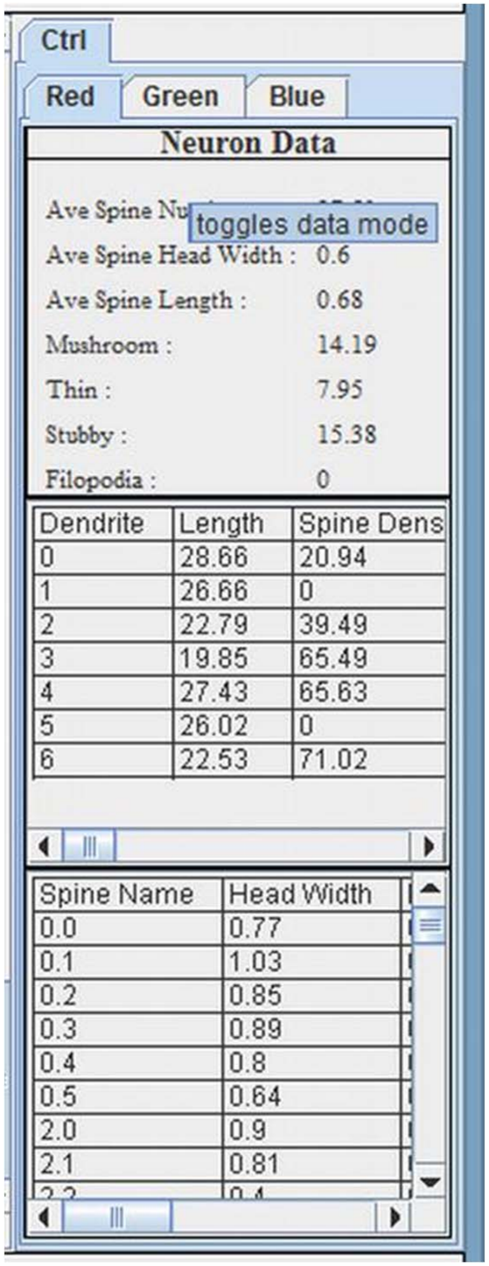

Generic Regions

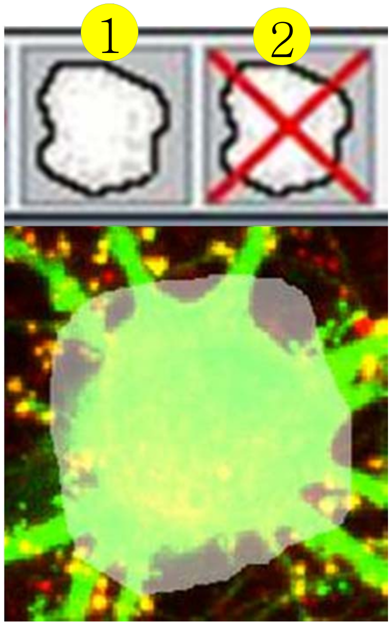

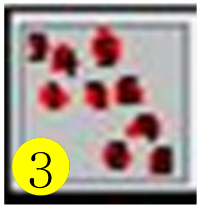

Add Generic Region

Clicking on Button (1) or pressing F3 will activate “Add Generic Region” mode. The user will left click and hold down the left mouse button to trace the desired region. Releasing the mouse button will finalize the region. This mode will stay active until the user double clicks on the image. After tracing, the average intensity per image will be automatically calculated. Changing the threshold settings will require Button (3) to be pressed to update the calculation.

Remove Generic Region

Clicking on Button (2) or pressing F4 will activate “Remove Generic Region” mode. Left clicking on any cell body object will permanently delete it.

Generic Region Data

Data for each color is displayed in the data pane. Data is averaged per image or displayed individually. Integrated intensity is the sum of pixel intensities for all pixels (above threshold) within the designated area of the region. Ave intensity is the integrated intensity divided by the area of the region.

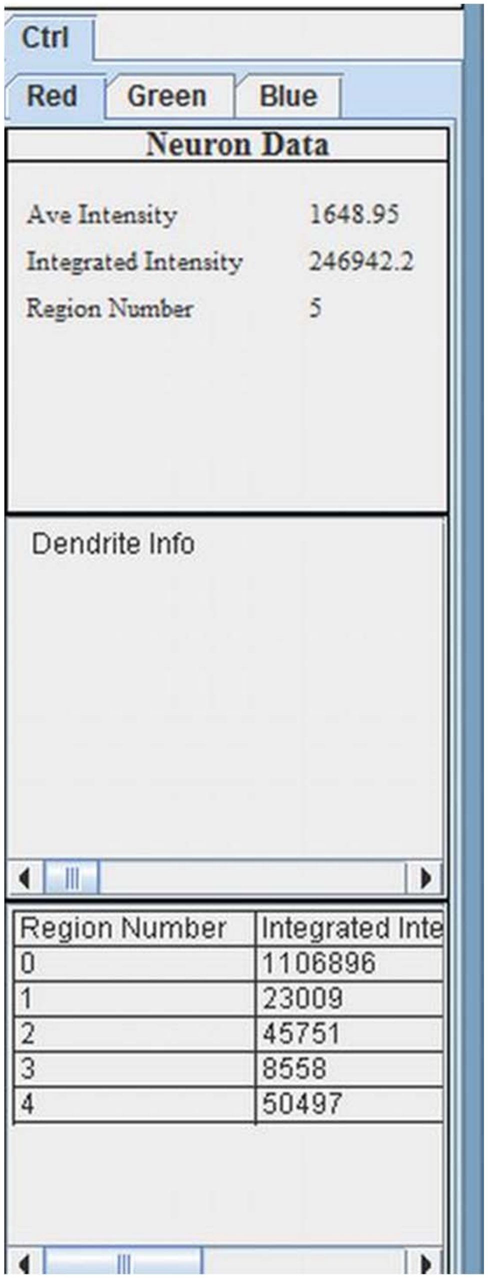

Whole Image Analysis

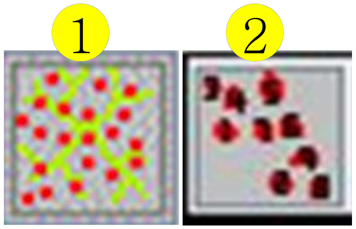

Add Whole Image Analysis Region
Clicking on Button (1) or pressing F12 will add a “dendrite” object that encompasses the entire width so the entire image can be analyzed at once. Like dendrite objects, the image must be thresholded for puncta to be detected. The length of this region will be 0 and cannot be changed. Puncta Density(A) = Puncta number / Area. Clicking on Button (2) will recalculate puncta density.

Remove Generic Region
The region can be removed by right clicking and selecting “Remove Dendrite”

Generic Region Data
Data for each color is displayed in the data pane. Data is averaged per image or displayed individually. Integrated intensity is the sum of pixel intensities for all pixels (above threshold) within the designated area of the region. Ave intensity is the integrated intensity divided by the area of the region. Puncta Density(L) will be infinite due to length 0. Puncta Density (A) is Puncta number divided by the area. Puncta Intensity / Length is infinite. Puncta Intensity / Area is the Integrated intensity divided by the area.

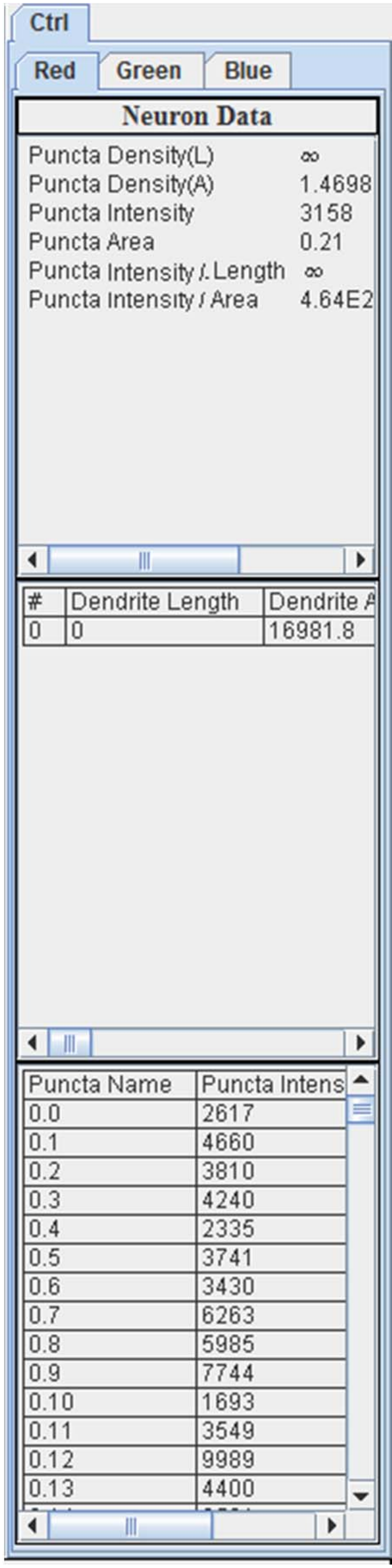

Right Click Options for Image Panel

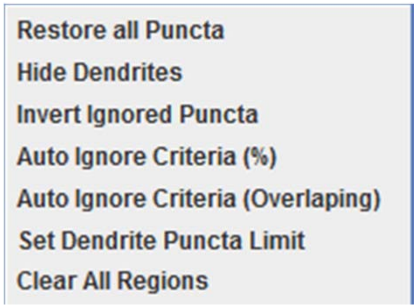

Restore all Puncta
Makes all puncta visible.

Show/Hide Dendrites
Makes all objects Visible/Invisible

Invert Ignored Puncta
Makes Ignored puncta visible and sets visible puncta to Ignored

Auto Ignore Criteria (%)
(1)Auto Ignores puncta based on percent overlap (area) with chosen thresholded color.

Auto Ignore Criteria (Overlaping)
(2) Auto Ignores puncta based on overlap with non thresholded color.

Set Dendrite Puncta Limit
(3) Auto Ignores all puncta smaller than set size. (Must be $\geq$ 4)

Clear All Regions
All regions are permanently removed.

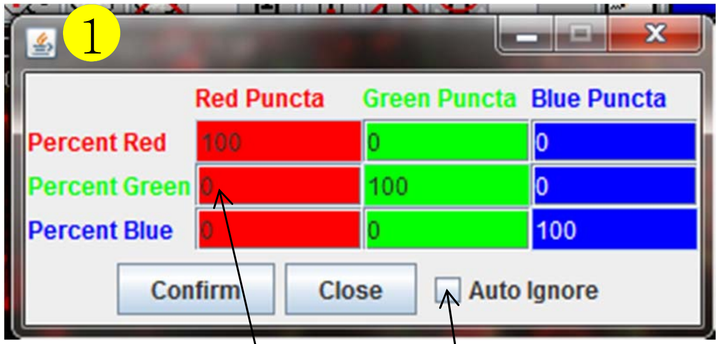

A value of 50 will remove any red puncta that overlaps (thresholded) green by 50%.

Will apply set criteria to each new region object added automatically for each new image analyzed

Check to remove Red puncta that overlap with green (if checked) or blue (if checked)

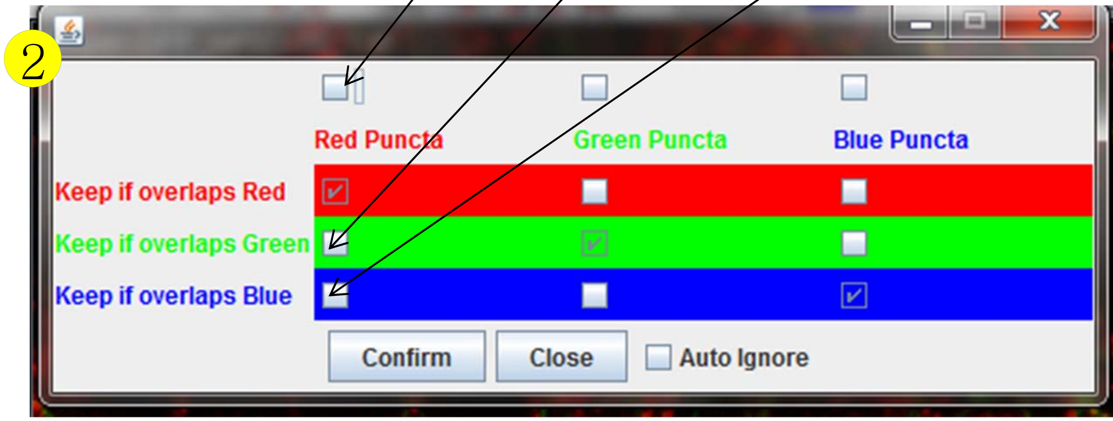

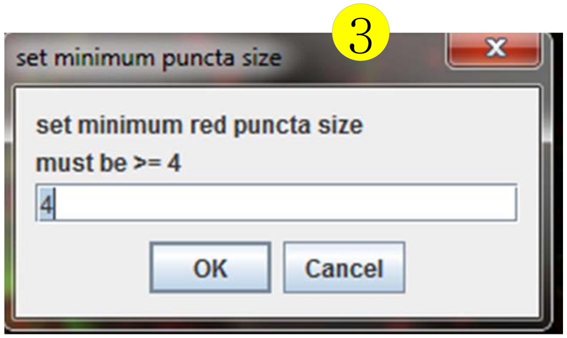

Supplement: S1 Text — SynPAnal User Manual. (PDF) [file pone.0115298.s001.pdf]
